# Supplementary figures and images for: Is ICE hot? A genomic comparative study reveals integrative and conjugative elements as “hot” vectors for the dissemination of antibiotic resistance genes
Source: mSystems. 2023 Nov 30;8(6):e00178-23. doi: 10.1128/msystems.00178-23 (PMC10734551; doi:10.1128/msystems.00178-23)

**A**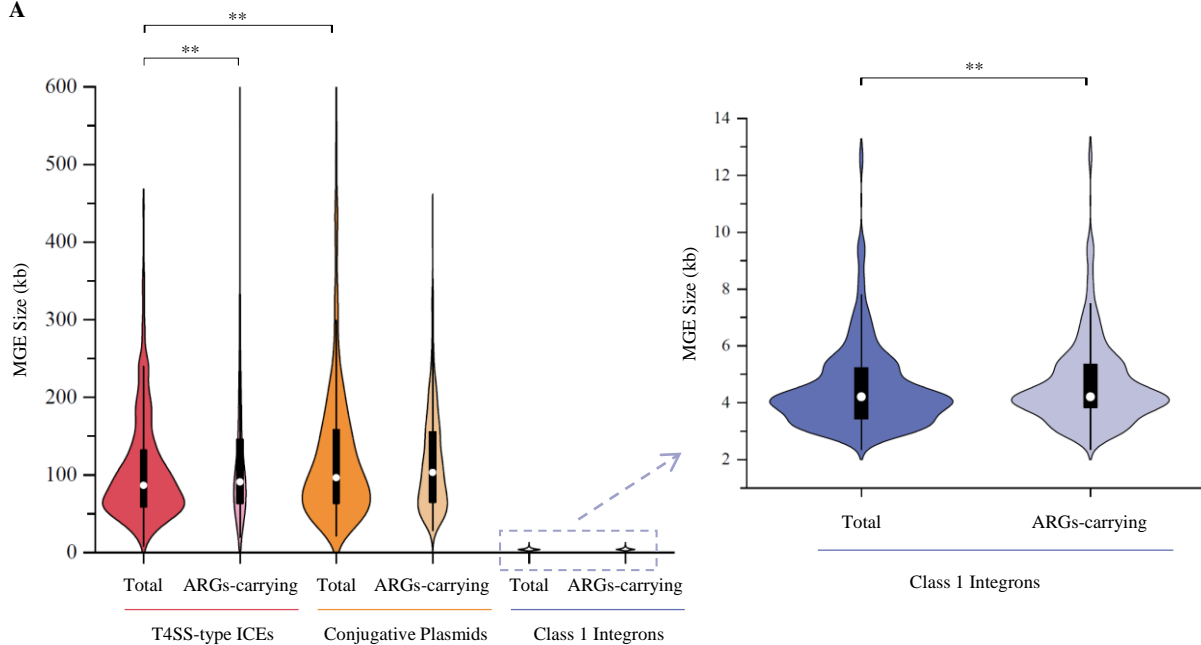**B**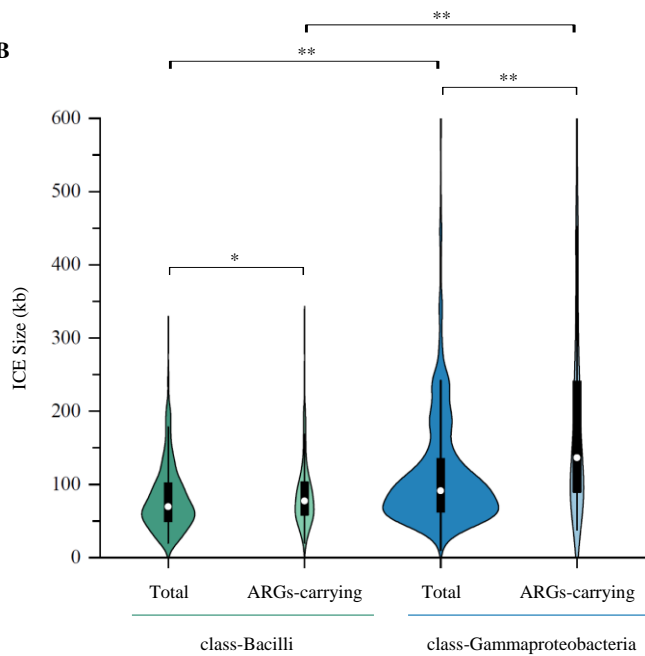

Supplement: Figure S1 — Size distribution of the three MGEs and their ARG-carrying ones (A), as well as T4SS-type ICEs across the two major classes—Bacilli and Gammaproteobacteria (B). [file msystems.00178-23-s0001.pdf]

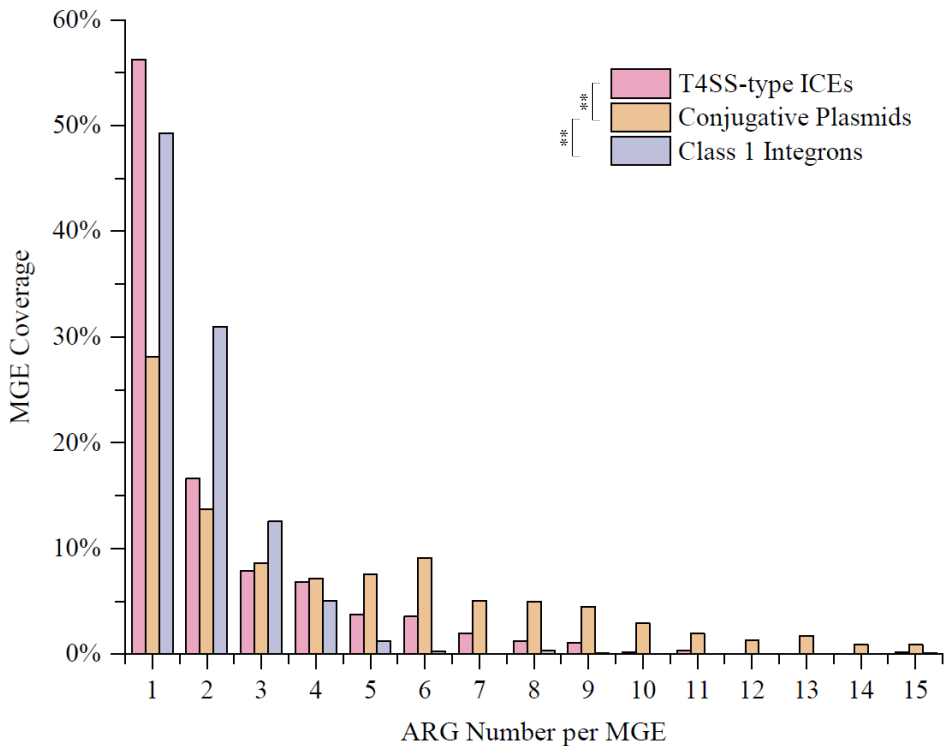

Supplement: Figure S2 — Coverage distribution of the three MGEs encoding different ARG numbers among their total ARG-carrying ones correspondingly. [file msystems.00178-23-s0002.pdf]

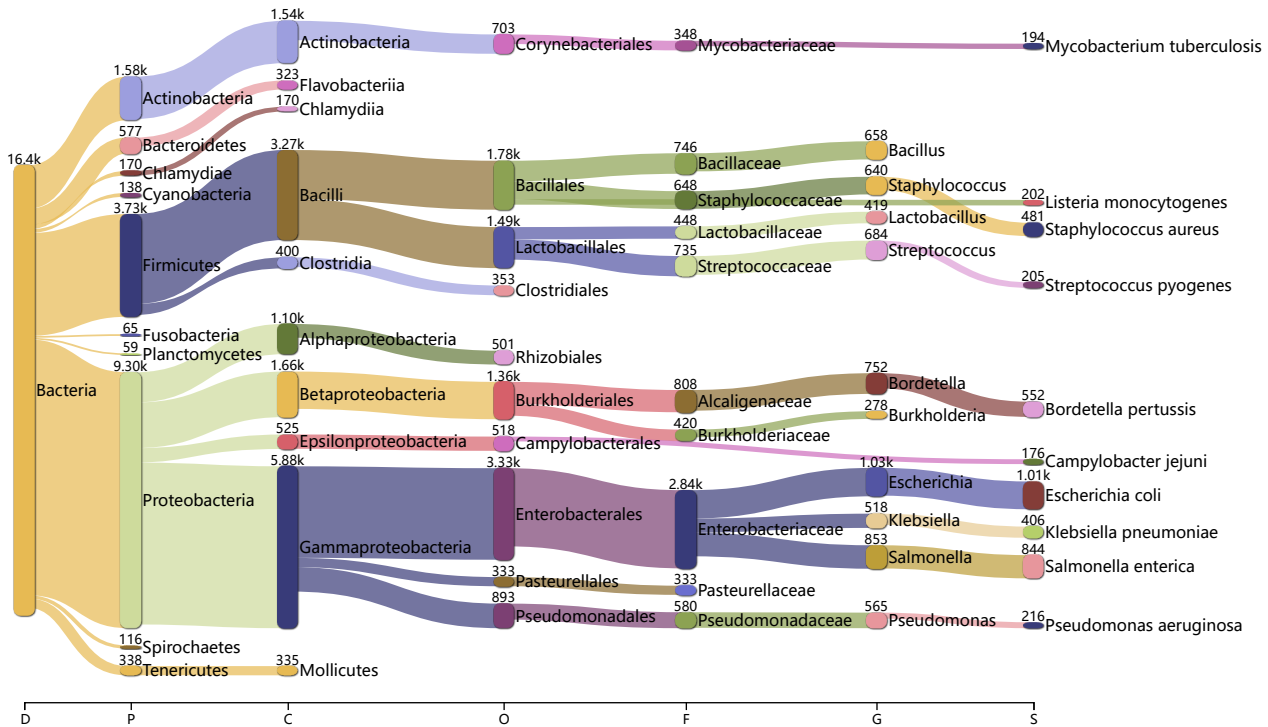

Supplement: Figure S3 — Phylogenetic distribution of the NCBI bacterial complete genome database (based on genome number). [file msystems.00178-23-s0003.pdf]

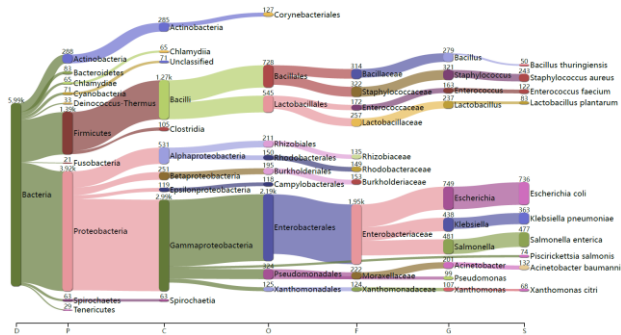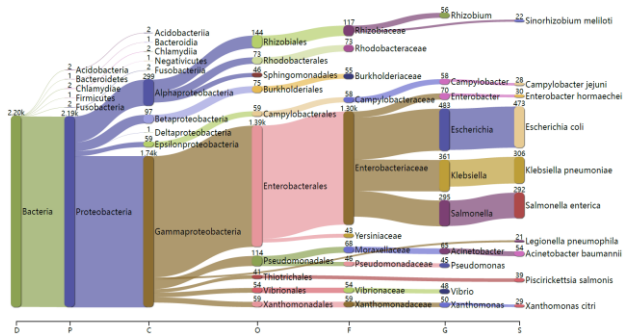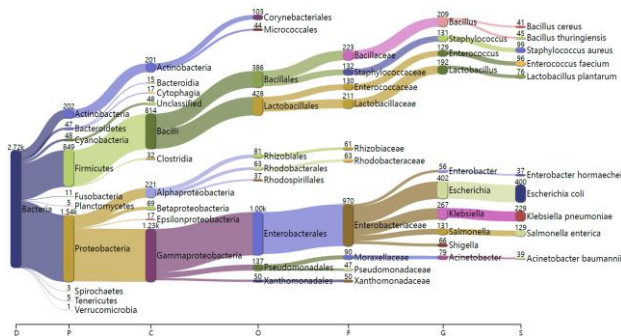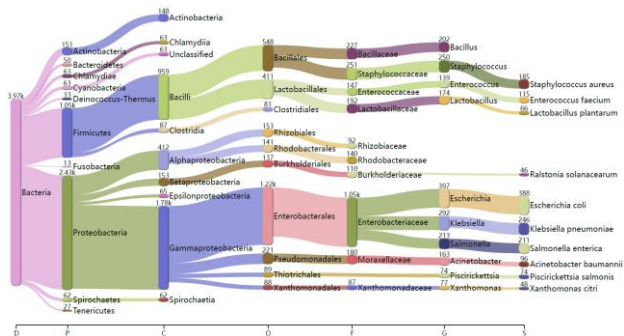

Supplement: Figure S4 — Phylogenetic distribution of bacteria hosting the total 14,813 plasmids as well as their three categories—conjugative, mobilizable, and non-mobilizable plasmids (based on genome number). [file msystems.00178-23-s0004.pdf]

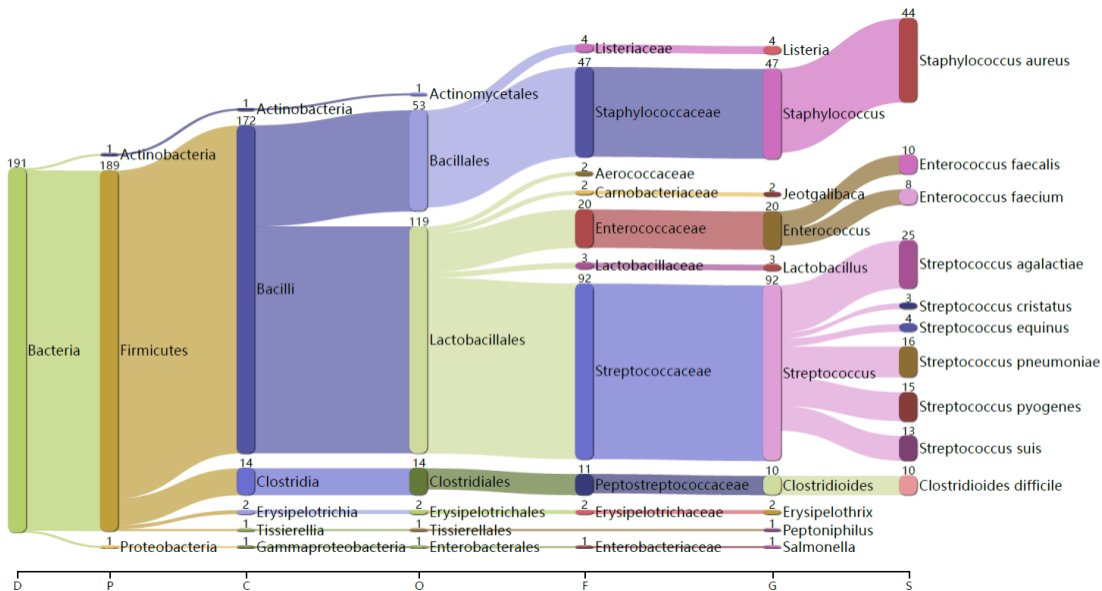

Supplement: Figure S5 — Phylogenetic distribution of bacteria hosting the T4SS-type ICEs encoding tetM resistant to tetracycline (based on genome number). [file msystems.00178-23-s0005.pdf]

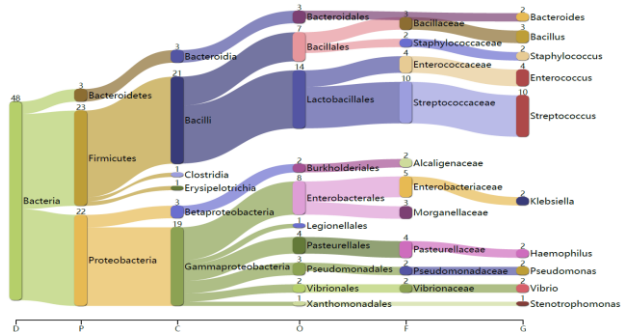

T4SS-type ICEs

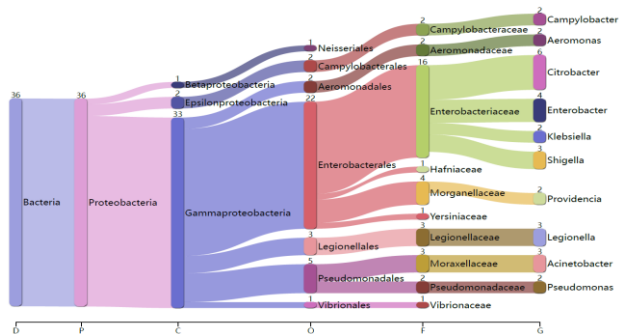

Conjugative Plasmids

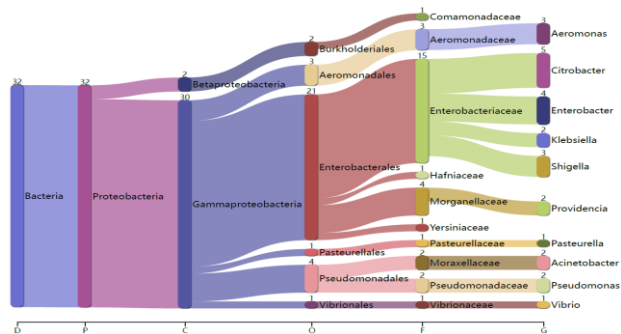

Class 1 Integrons

Supplement: Figure S6 — Phylogenetic distribution of pathogenic species hosting the three ARG-carrying MGEs (based on species number). [file msystems.00178-23-s0006.pdf]
