# Supplementary material for: Is ICE hot? A genomic comparative study reveals integrative and conjugative elements as “hot” vectors for the dissemination of antibiotic resistance genes
Source: mSystems. 2023 Nov 30;8(6):e00178-23. doi: 10.1128/msystems.00178-23 (PMC10734551; doi:10.1128/msystems.00178-23)
Supplement: Figure S7 — Phylogenetic distribution of bacteria hosting the two MGEs that carry ARGs and VFs—T4SS-type ICEs (A) and conjugative plasmids (B) (based on genome number). [file msystems.00178-23-s0007.pdf]

A

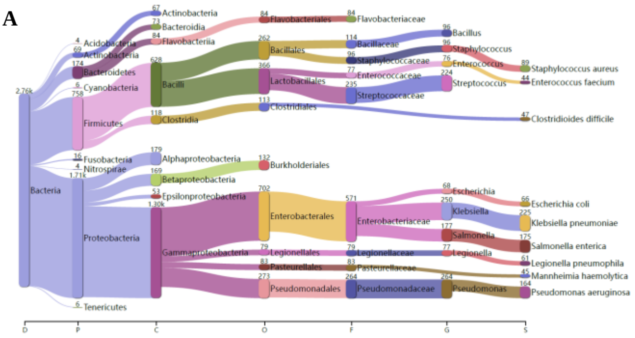

Total T4SS-type ICEs

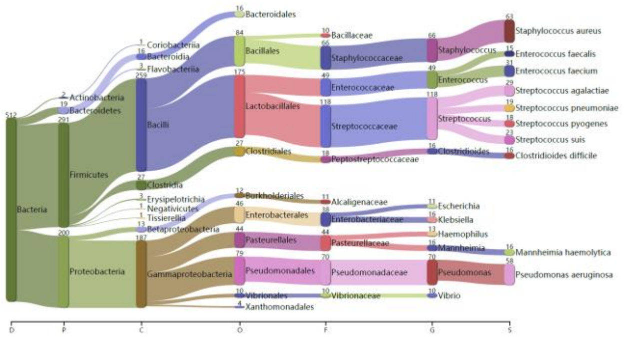

ARGs-carrying T4SS-type ICEs

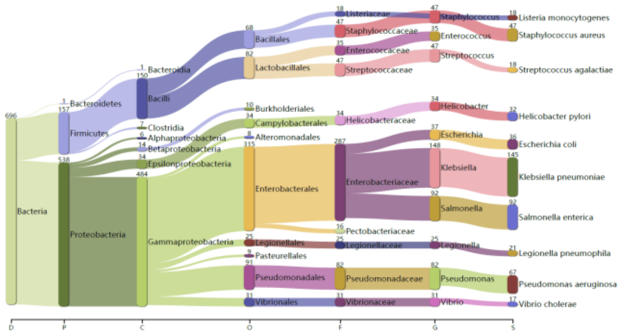

VFs-carrying T4SS-type ICEs

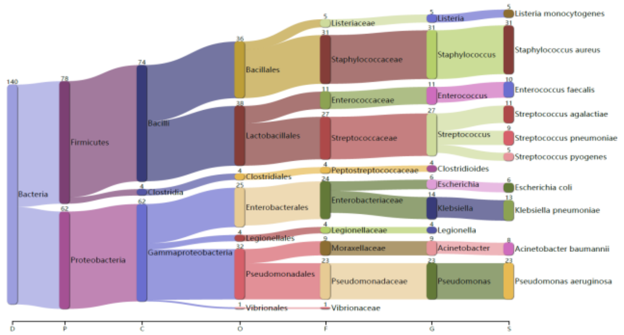

T4SS-type ICEs with Co-occurrence of ARGs and VFs

B

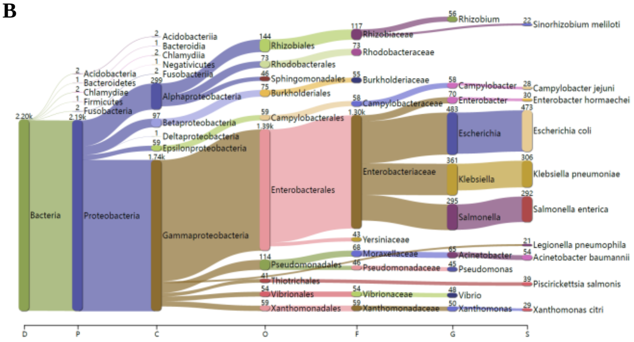

Total Conjugative Plasmids

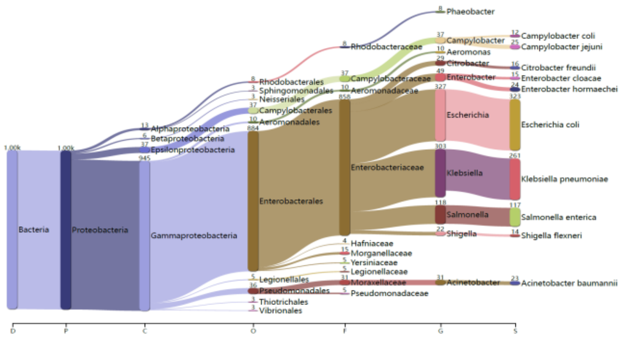

ARGs-carrying Conjugative Plasmids

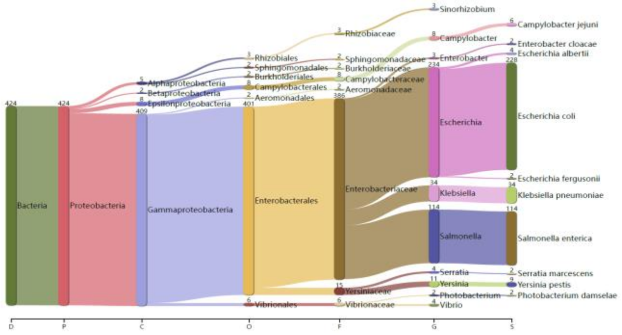

VFs-carrying Conjugative Plasmids

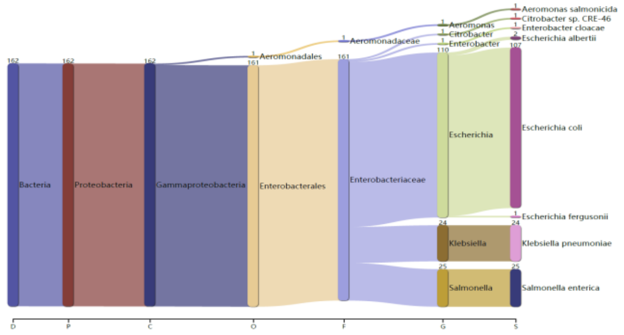

Conjugative Plasmids with Co-occurrence of ARGs and VFs
