# Supplementary material for: Is ICE hot? A genomic comparative study reveals integrative and conjugative elements as “hot” vectors for the dissemination of antibiotic resistance genes
Source: mSystems. 2023 Nov 30;8(6):e00178-23. doi: 10.1128/msystems.00178-23 (PMC10734551; doi:10.1128/msystems.00178-23)
Supplement: Supplemental legends — Legends for supplemental figures and tables. [file msystems.00178-23-s0008.docx]

**Supplemental Material List**

(1) Supplementary Figures contain:

Supplementary Figure S1. Size distribution of the three MGEs and their ARGs-carrying ones (A), as well as T4SS-type ICEs across the two major classes – *Bacilli* and *Gammaproteobacteria* (B). p-value was evaluated via B-H corrected Mann-Whitney test, and * means p-value < 0.05 while ** means p-value < 0.01.

Supplementary Figure S2. Coverage distribution of the three MGEs encoding different ARG numbers among their total ARGs-carrying ones correspondingly. p-value was evaluated via B-H corrected Mann-Whitney test, and ** means p-value < 0.01.

Supplementary Figure S3. Phylogenetic distribution of the NCBI bacterial complete genome database (based on genome number).

Supplementary Figure S4. Phylogenetic distribution of bacteria hosting the total 14,813 plasmids as well as their three categories – conjugative, mobilizable, and non-mobilizable plasmids (based on genome number).

Supplementary Figure S5. Phylogenetic distribution of bacteria hosting the T4SS-type ICEs encoding *tetM* resistant to tetracycline (based on genome number).

Supplementary Figure S6. Phylogenetic distribution of pathogenic species hosting the three ARGs-carrying MGEs (based on species number).

Supplementary Figure S7. Phylogenetic distribution of bacteria hosting the two MGEs that carry ARGs and VFs – T4SS-type ICEs (A) and conjugative plasmids (B) (based on genome number).

(2) Supplementary Tables contain:

Supplementary Table S1. Detailed metadata (assembly accession number, taxonomic lineage, and potential pathogenicity) of the total 16,364 bacterial complete genomes used in this study.

Supplementary Table S2. Basic information of the three MGE groups extracted from NCBI bacterial complete genome database.

Supplementary Table S3. Detailed ARG profiles (ARG proportion and MGE coverage) of the three MGE groups.

Supplementary Table S4. Detailed ARG profiles (ARG proportion and MGE coverage) of T4SS-type ICEs across the two major classes – *Bacilli* and *Gammaproteobacteria*.

Supplementary Table S5. Detailed ARG profiles (ARG proportion and MGE coverage) of the three MGE groups located on pathogens and non-pathogens.

Supplementary Table S6. Basic information (ARG coverage, ARG type number, and ARG subtype number) of the three MGE groups hosted by pathogenic species.

Supplementary Table S7. Detailed ARG profiles (ARG proportion and MGE coverage) of the three MGE groups hosted by their dominant pathogenic species.

Supplementary Table S8. Detailed VF profiles (VF proportion and MGE coverage) of the two MGE groups – T4SS-type ICEs and conjugative plasmids.

Supplementary Table S9. Detailed VF profiles (VF proportion and MGE coverage) of the T4SS-type ICEs and conjugative plasmids with typical co-occurrence patterns of ARGs and VFs hosted by their dominant pathogenic species.

Supplementary Table S10. Detailed ARG profiles (ARG proportion and MGE coverage) of the class 1 integrons embedded in T4SS-type ICEs and conjugative plasmids.
